# Prediction and Modeling of Neuropsychological Scores in Alzheimer’s Disease Using Multimodal Neuroimaging Data and Artificial Neural Networks

**DOI:** 10.3389/fncom.2021.769982

**Published:** 2022-01-06

**Authors:** Seyed Hani Hojjati, Abbas Babajani-Feremi, the Alzheimer’s Disease Neuroimaging Initiative

**Affiliations:** ^1^Quantitative Neuroimaging Laboratory, Brain Health Imaging Institute, Department of Radiology, Weill Cornell Medicine, New York, NY, United States; ^2^Department of Neurology, Dell Medical School, The University of Texas at Austin, Austin, TX, United States; ^3^Department of Neurosurgery, Dell Medical School, The University of Texas at Austin, Austin, TX, United States; ^4^Magnetoencephalography Laboratory, Dell Children’s Medical Center, Austin, TX, United States

**Keywords:** Alzheimer’s disease, neuropsychological scores, structural MRI, FDG-PET, artificial neural networks (AANs)

## Abstract

**Background:** In recent years, predicting and modeling the progression of Alzheimer’s disease (AD) based on neuropsychological tests has become increasingly appealing in AD research.

**Objective:** In this study, we aimed to predict the neuropsychological scores and investigate the non-linear progression trend of the cognitive declines based on multimodal neuroimaging data.

**Methods:** We utilized unimodal/bimodal neuroimaging measures and a non-linear regression method (based on artificial neural networks) to predict the neuropsychological scores in a large number of subjects (*n* = 1143), including healthy controls (HC) and patients with mild cognitive impairment non-converter (MCI-NC), mild cognitive impairment converter (MCI-C), and AD. We predicted two neuropsychological scores, i.e., the clinical dementia rating sum of boxes (CDRSB) and Alzheimer’s disease assessment scale cognitive 13 (ADAS13), based on structural magnetic resonance imaging (sMRI) and positron emission tomography (PET) biomarkers.

**Results:** Our results revealed that volumes of the entorhinal cortex and hippocampus and the average fluorodeoxyglucose (FDG)-PET of the angular gyrus, temporal gyrus, and posterior cingulate outperform other neuroimaging features in predicting ADAS13 and CDRSB scores. Compared to a unimodal approach, our results showed that a bimodal approach of integrating the top two neuroimaging features (i.e., the entorhinal volume and the average FDG of the angular gyrus, temporal gyrus, and posterior cingulate) increased the prediction performance of ADAS13 and CDRSB scores in the converting and stable stages of MCI and AD. Finally, a non-linear AD progression trend was modeled to describe the cognitive decline based on neuroimaging biomarkers in different stages of AD.

**Conclusion:** Findings in this study show an association between neuropsychological scores and sMRI and FDG-PET biomarkers from normal aging to severe AD.

## Introduction

Alzheimer’s disease (AD) is a neurodegenerative disease with progressive loss of memory and other functions that can be recognized using neuropsychological evaluation (Schmidtke and Hüll, 2002). Different domains of brain functions can be assessed through neuropsychological tests such as the Clinical Dementia Rating Sum of Boxes (CDRSB), Alzheimer’s Disease Assessment Scale Cognitive13 (ADAS13), Mini-Mental State Exam (MMSE), and Rey Auditory Verbal Learning Test (RAVLT) (Crane et al., 2012). Neuropsychological tests and neuroimaging data have been increasingly used for identifying patients with AD and mild cognitive impairment (MCI). MCI is typically considered a stage between healthy aging and AD (Ramakers et al., 2008; Duc et al., 2020). Some patients with MCI progress to AD, MCI converter (MCI-C), and the rest of them do not progress to AD, MCI non-converter (MCI-NC) (Gomez-Sancho et al., 2018; Shen et al., 2018). Considering that MCI-C and MCI-NC patients are in an intermediate stage between healthy aging and AD, it is challenging to identify robust and reliable biomarkers for prediction of progression of these patients to AD.

AD is a complex brain disease and integration of different neuropsychological tests is needed to adequately identify the evidence of dementia. It has also been shown in previous studies that neuropsychological scores are intrinsically correlated with each other (Van Der Maas et al., 2006; Tosi et al., 2020), and, thus, individuals with a healthy score in one neuropsychological test are more likely to achieve a healthy score in other tests. To efficiently identify the early stage of AD, it is critical to find the relationship and association between different neuropsychological tests.

It has been shown that AD starts years before any appearance of symptoms, and alterations in the brain structural and network characteristics occur while neuropsychological scores are still normal (Sperling et al., 2014). In recent years, several studies focused on diagnostic criteria for the early stage of AD based on neuroimaging biomarkers (Moradi et al., 2015; Hojjati et al., 2018, Hojjati et al., 2019). Neuroimaging-based diagnosis of AD [e.g., using structural magnetic resonance imaging (sMRI) and fluorodeoxyglucose (FDG) from positron emission tomography (PET)] can identify biomarkers that are sensitive to changes in the brain in the early stages of the disease (Eskildsen et al., 2013; Pagani et al., 2015; Hojjati et al., 2019; Tabarestani et al., 2020). Several neuroimaging studies have shown that the neuroimaging biomarkers may be more capable than the neuropsychological tests in identifying the early stage of AD (Leung et al., 2010; Gao et al., 2015; Hojjati et al., 2018; Lu et al., 2018).

Recent advances in the neurobiology of AD suggested that AD is a multifactorial and heterogeneous disease that cannot be explained by a single biomarker (Jack et al., 2018). Over the past decades, several projects have been funded to collect data from large cohorts of older adults. However, less effort has been made to implement integrative methods for aggregating data across modalities and capture the heterogeneity of AD. Previous studies utilized neuroimaging biomarkers, mostly in a single-modality approach, to find a relationship between neuropsychological tests and AD risk factors (Frisoni et al., 2002, 2010). It has been demonstrated that the trajectories of neuroimaging biomarkers for prediction of AD are complex and have a non-linear trend due to the interactions with age (Sabuncu et al., 2011). Hence, a non-linear relationship between neuropsychological scores and neuroimaging features should be considered. In the current study, we used artificial neural networks (ANNs) to investigate a non-linear model for AD progression trend based on neuropsychological scores. ANNs have been successfully utilized for various brain imaging applications (Savioz et al., 2009; Choi et al., 2020; Wen et al., 2020) and have gained increasing interest in diverse applications, such as classification, speech recognition, age modeling, modeling and forecasting extreme events, and even face recognition (Cole et al., 2017; Tuan Tran et al., 2017; Duc et al., 2020; Chowdhury et al., 2021). Moreover, ANN was utilized to provide an effective method for early diagnosis of AD (Wang et al., 2019). In particular, ANNs were shown to be effective in predicting progression from healthy cognition to AD (Albright and Alzheimer’s Disease Neuroimaging Initiative, 2019), and this predictive ability of ANNs outperformed that of a linear model (Grossi et al., 2007).

In this study, we investigated finding neuroimaging biomarkers as inputs features of an ANN to accurately model target neuropsychological scores (e.g., ADAS13) in a wide range of cognitive impairments, from healthy aging to severe AD, in four groups of subjects (healthy controls (HC), MCI-NC, MCI-C, and AD). By including data from MCI-C patients in our study, we investigated the accuracy of this model in a challenging condition by using their data in the baseline (when they showed a MCI) before they progressed to AD in the next several months or years. There are a few studies that proposed methods to predict and model the neuropsychological scores (Apostolova et al., 2006; Ferrarini et al., 2008). Duc et al. (2020) predicted the Mini-Mental State Exam (MMSE) score based on resting-state functional MRI using various regression methods (e.g., support vector regression and bagging-based ensemble regression) and utilized the 3-D convolutional neural network to classify two groups of subjects (HC and AD). In Ferrarini et al. (2008), the authors investigated an association between the atrophy in periventricular structures and cognitive impairment in MCI and AD, as estimated by the MMSE score. Moradi et al. (2017) utilized whole-brain gray matter density maps for predicting the RAVLT Immediate and RAVLT Percent Forgetting scores based on a machine learning approach. They reported *R* = 0.50 and *R* = 0.43 correlation between the estimated and observed RAVLT Immediate and RAVLT Percent Forgetting, respectively. Ito et al. (2011) attempted to fit a linear model for the longitudinal ADAS scores by including age, apolipoprotein 34 [APOE 34] genotype, gender, family history of AD, years of education, and baseline severity as inputs of the model. Their results showed that AD progression increased with baseline severity while age, APOE 34 genotype, and gender also influenced this progression. In another study, Liu et al. (2019) proposed supervised densely connected neural network methods to predict neuropsychological scores (i.e., CDRSB and MMSE) in three groups (HC, MCI, and AD). They utilized the landmark detection algorithm and found future multiple clinical scores in four different time-points using single modal neuroimaging of baseline MRI data.

This is the first study to our knowledge that attempted to model the progression trend of neuropsychological scores using a non-linear ANN approach based on bi-modal neuroimaging data in a large sample size (*n* = 1143) and four groups of subjects (HC, MCI-NC, MCI-C, and AD). In the current study we: (1) applied a machine learning approach based on ANN to predict ADAS13 and CDRSB scores (as the target scores) from normal aging to AD using neuroimaging biomarkers and other neuropsychological scores; (2) compared the performances of sMRI and PET in single- and bi-modal approaches to find the best neuroimaging biomarkers for predicting the target neuropsychological scores; (3) evaluated the ability of the best neuroimaging biomarkers in predicting the ADAS13 score in stable (i.e., MCI-C) and converting (i.e., MCI-NC) stages of AD; and (4) compared the progression trend of sMRI and PET biomarkers based on ADAS13 to find the prediction power of these biomarkers in different stages of AD. It is noteworthy that we chose the CDRSB and ADAS13 as the target tests because of their sensitivity to the assessment of the severity of AD progression and general cognitive functions (Kueper et al., 2018).

## Materials and Methods

### Overall Procedure

**FIGURE 1:**
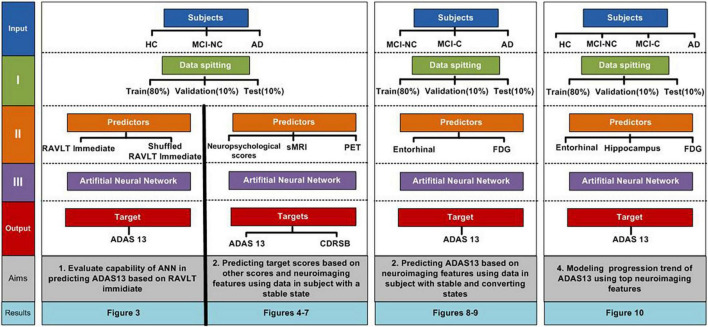
Overall procedures of this study.

The preprocessed structural MRI (T1-weighted images), PET, and neuropsychological data of 1143 subjects from The Alzheimer’s Disease Prediction Of Longitudinal Evolution (TADPOLE) challenge^1^ were used in this study (Figure 1; Marinescu et al., 2018). In the first part of this study, we applied randomization test in predicting ADAS13 based on the RAVLT immediate to demonstrate feasibility of using ANN algorithm for this prediction. The subjects were randomly split to train, validation, and test sets (Figure 1) and the performances of ANN for prediction of ADAS13 were evaluated based on real and random shuffled (across subjects) RAVLT immediate data.

**FIGURE 2:**
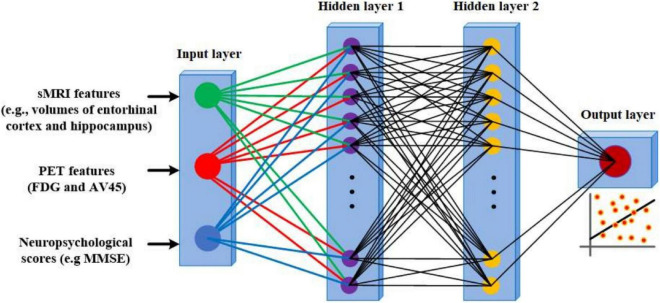
The schematic diagram of the architecture of ANN used in this study.

In the second part of this study, we used an ANN to predict the ADAS13 and CDRSB based on other neuropsychological scores and neuroimaging data. Three stable groups of subjects were used in this part: HC, patients with AD, and patients with MCI-NC whose diagnosis did not change in their follow-up visits. We excluded the MCI-C patients in this part of our study because their state was not stable, as these patients had multiple conversions and revisions from MCI to AD and vice-versa in their follow-up visits (Hojjati et al., 2018). Another reason for excluding MCI-C patients was to use them as an independent test-set to evaluate performance of the trained ANN model based on other three groups of subjects. The data of 951 subjects (341 HC, 393 MCI-NC, and 217 AD) were used in the first part of this study. We utilized an ANN with two hidden layers (Figure 2) to predict the target scores (ADAS13 or CDRSB). The sMRI and PET features (single-modal or bimodal) and other neuropsychological scores (e.g., MMSE and RAVLT) were used as input features of the ANN. Subjects were randomly split 500 times into training (80%), cross-validation (10%), and test (10%) sets, and performance of the ANN models in cross-validation and test sets were evaluated.

In the third part of this study, we investigated the ability of the best neuroimaging biomarkers for predicting ADAS13 in MCI and AD using data of 748 subjects (339 MCI-NC, 192 MCI-C, and 217 AD). It is noteworthy that predicting the cognitive scores of MCI-C patients is a challenging task since we used their baseline neuroimaging data, and they converted to AD in their future follow-up visits. In addition, the MCI-C group had heterogeneity in their conversion time to AD from 6 to 36 months. Therefore, the MCI-C patients who converted to AD in a longer time (e.g., at 36 months) after baseline may have similar brain functional and structural characteristics compared to the MCI-NC patients. On the other hand, the MCI-C patients who converted to AD in a shorter time (e.g., at 6 months) after baseline may have similar brain characteristics compared to AD patients. To test our model in a challenging condition, we utilized a subset of MCI-NC patients (80%; *n* = 271) and a subset of AD patients (80%; *n* = 173) to train and cross-validate an ANN using the neuroimaging biomarkers as input features and ADAS13 as a target of prediction. Subsequently, we tested the ANN using the following three test sets: untrained MCI-NC patients (20%; *n* = 68 patients), untrained AD patients (20%; *n* = 44), and all MCI-C patients (100%, *n* = 192).

In the last part of this study, we used the best neuroimaging biomarkers for each modality (sMRI and PET) to fit non-linear progression trends from normal aging to AD for ADAS13. All four groups of subjects (341 HC, 192 MCI-C, 393 MCI-NC, and 217 AD) were included in this part. The ANN and statistical analyses were implemented in MATLAB (Mathworks, Natick, MA, United States).

### Neuroimaging and Neuropsychological Data

We used a subset of data provided by TADPOLE challenge. The aim of this challenge was to compare the performance of different algorithms for predicting the future evolution of individuals at risk for AD. Data for this challenge were obtained from the Alzheimer’s Disease Neuroimaging Initiative (ADNI) database^2,3^. In the current study, we used neuropsychological scores and neuroimaging data (MRI and PET) in 217 AD (73.2 ± 7.5 years; 124 male), 192 MCI-C (73.5 ± 7.1 years; 111 male), 393 MCI-NC (74.1 ± 7.5 years; 232 male), and 341 HC (74.8 ± 5.7 years; 157 male) subjects from the TADPOLE challenge database (Table 1).

**TABLE 1:** Demographic and clinical information of four groups of subjects.

	HC	MCI-NC	MCI-C	AD
Number of subjects	341	393	192	217
Male/Female	157/184	232/161	111/81	124/93
Age, year (mean ± SD)	74.85 ± 5.66	74.10 ± 7.52	73.49 ± 7.07	72.24 ± 7.54
ADAS13 score (mean ± SD)	8.46 ± 4.36	14.36 ± 6.46	20.41 ± 6.41	30.38 ± 8.37
CDRSB score (mean ± SD)	0.06 ± 0.30	1.39 ± 0.88	1.92 ± 0.97	4.47 ± 1.71
MMSE score (mean ± SD)	29.04 ± 1.13	27.80 ± 1.96	27.05 ± 1.88	23.10 ± 2.20
RAVLT immediate (mean ± SD)	46.02 ± 9.86	36.81 ± 10.48	28.33 ± 7.87	22.30 ± 7.43
RAVLT learning (mean ± SD)	6.06 ± 2.17	4.63 ± 2.59	2.99 ± 2.29	1.74 ± 1.64
RAVLT forgetting (mean ± SD)	3.81 ± 2.76	4.43 ± 2.59	5.06 ± 2.07	4.41 ± 1.78

#### Cognitive Assessments

Cognitive assessments in this study comprised different neuropsychological tests including CDRSB, MMSE, ADAS13, RAVLT Immediate, RAVLT Learning, and RAVLT Forgetting. The CDRSB is the global clinical measure that represents six cognitive areas (i.e., memory, orientation, judgment, community affairs, home and hobbies, and personal care) (Morris, 1991; Rogers et al., 2000). The MMSE is a cognitive function test of orientation, attention, memory, language, and visual-spatial skills (Folstein et al., 1975). The ADAS13 is a measure of multiple cognitive domains including memory, language, praxis, orientation, executive functioning, and functional ability (Skinner et al., 2012). The RAVLT is sensitive to verbal memory deficits and consists of presenting 15 words (a trial) across five consecutive trials (Rey, 1958). The participants are instructed to recall as many as words they remember after each trial. The RAVLT Immediate is the sum of scores of 5 trials. The RAVLT Learning is the score of Trial 5 minus the score of Trial 1. The RAVLT Forgetting is the score of Trial 5 minus the score of the delayed recall trial (Trial 6). In Trial 6, the participant is asked to recall the words from the first list after presenting a list of new 15 words.

#### Neuroimaging Data

For sMRI biomarkers in the current study, we included the total intracranial volume (ICV) and the volumes of the following five ROIs as input features of the ANN: the middle temporal gyrus (MIDTEMP), hippocampus, entorhinal cortex, ventricles, and fusiform gyrus. We also used fluoro-deoxyglucose (FDG) and AV45 PET as input features of the ANN models. The FDG-PET measures cell metabolism in the brain areas where the affected areas by AD show reduced metabolism. The AV45 PET measures density of amyloid-beta (Aβ) protein in the brain, where improper construction of Aβ can lead to AD. As provided by the TADPOLE database, the average FDG of angular gyrus, temporal gyrus, and posterior cingulate and the average AV45 of frontal, anterior cingulate, precuneus, and parietal cortex were used as the FDG and AV45 input features in our ANN model. To increase the reliability of this study, we worked to select the maximum number of HC, MCI-NC, MCI-C, and AD subjects having both sMRI and PET features.

### Data Preprocessing

The neuroimaging measures (i.e., sMRI and PET) used in the current study were provided by the TADPOLE database and were extracted from individual subjects’ brains after normalizing to the “standard” brain space (standardization) (Marinescu et al., 2018). Normalization removes the effects of inter-subject anatomical variability due to differences in brain size and shape. The T1-weighted MRI of all subjects was processed by ADNI-MRI-preprocessing pipelines for gradient non-linearity correction, B1 non-uniformity correction, and peak sharpening^4^. After the preprocessing steps, the volumes of different brain areas were extracted using the Freesurfer cross-sectional and longitudinal pipelines (Reuter et al., 2012). PET images (FDG and AV45), which had a series of dynamic frames, were processed by ADNI-PET-preprocessing pipelines consist of the following steps: frames co-registered, averaged across the dynamic range, standardized with respect to the orientation and voxel size, and smoothed using a uniform resolution with FWHM of 8 mm^5^. After preprocessing, the PET images were registered to corresponding MR images using the SPM software (Ashburner, 2009). From the registered and normalized PET images, standardized uptake value ratio (SUVR) measures for relevant regions of interest (ROI) were extracted. In addition to the spatial brain normalization and to address sensitivity of the ANN to feature scaling, we normalized each of the neuroimaging features across all subjects (*n* = 1143) using the Min-Max scaling method to shift and rescale them so that they ended up ranging between 0 and 1.

### Artificial Neural Network Fitting

**FIGURE 3:**
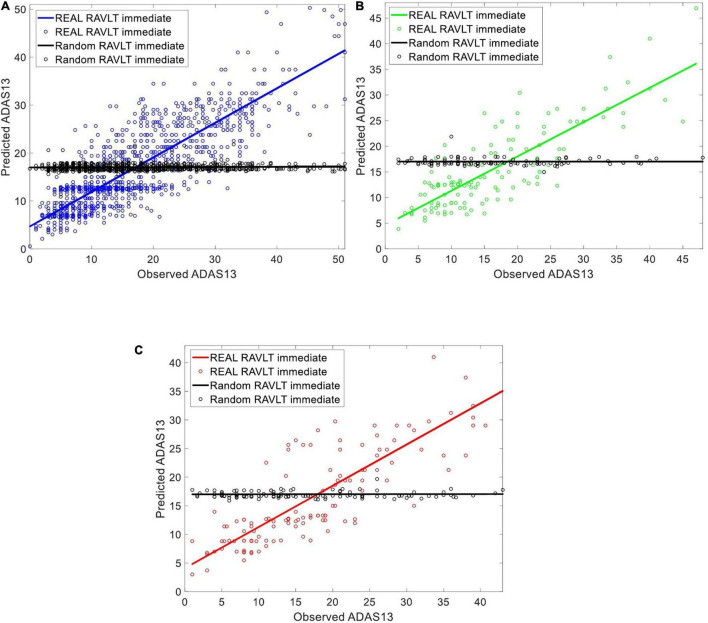
Evaluating the ability of the ANN in predicting ADAS13 based on RAVLT Immediate in subjects with a stable state (i.e., HC, MCI-NC, and AD) using a random shuffling approach where either real value of RAVLT Immediate or its random value by shuffling subjects (black color in the figure) were used for this prediction. The subplots are corresponding to **(A)** training set [80%] **(B)** validation set [10%], and **(C)** test set [10%] of the subjects in the three groups.

Artificial neural network (ANN) fitting is a logic-based method that uses a network architecture of interconnected hidden layers to model the relationship between input and output variables (Pao, 1989; Bullinaria, 2004). The ANN is an efficient machine learning algorithm that offers a number of advantages over other algorithms, including demanding less statistical training, capability to model complex non-linear relationships between dependent and independent variables, detecting interactions between predictor variables, and the accessibility of several training algorithms (Yegnanarayana, 2009). In the current study, we used the feed-forward multilayer perceptron (MLP) structure for the ANN with two hidden layers and 20 neurons based on experimental analysis (Figure 2). We observed that the complexity and processing time of the ANNs increased using more than 2 hidden layers and 20 neurons in each layer while performance of the ANNs did not improve substantially. The initial weights of ANN had random values between –1 and 1 and were initialized using a symmetric random weight function. The network was trained using the Levenberg–Marquardt learning algorithm (Fun and Hagan, 1996) and the sigmoidal tangent activation function (Zadeh et al., 2010). We used a learning rate of 0.001 and a maximum number of epochs of 1000 for generalization of the ANN training. The training of the ANN continued until generalization stops improving as specified by an increase in the mean square error (MSE) of the validation set for six iterations (validation pause). The MSE-observation and *R*-square were used to evaluate the performance of ANN fitting. The MSE is the average squared difference between the estimated output and target and was minimized as the loss-function during training and validation of the ANN. The *R*-square is the square of the correlation between the estimated output and target. We used the *R*-square in addition to the MSE in order to have a performance measure that is independent of the scale of data. We split data into training (80%), validation (10%), and test (10%) sets in this study and reported the performance of the ANN on the test set in the result section. Furthermore, the feasibility of prediction based on this ANN approach was verified by the random shuffling test (Figure 3).

## Results

### Capability of Artificial Neural Network in Predicting ADAS13 Based on Rey Auditory Verbal Learning Test

We evaluated the ability of the ANN in predicting ADAS13 based on RAVLT Immediate in subjects with a stable state (i.e., HC, MCI-NC, and AD) using a random shuffling approach. To this end, ADAS13 was predicted in two conditions where either value of RAVLT Immediate in all subjects or random values (by shuffling subjects) were used for this prediction. Considering that ADAS13 and RAVLT have overlaps over the different cognitive domains, we hypothesized that the ANN with real RAVLT Immediate as input feature can predict ADAS13 but random shuffling of this input feature cannot provide a meaningful prediction. Figure 3 shows the predicted and observed ADAS13 based on real and shuffled RAVLT Immediate. As expected, the ANN could not predict the ADAS13 with random shuffling values (*R*-square = 0.01 and RMSE = 116.42 based on test set), but the ADAS13 was predicted well with the real RAVLT Immediate values (*R*-square = 0.80 and RMSE = 33.10 based on test set).

### Predicting Target Neuropsychological Scores Based on Other Scores

**FIGURE 4:**
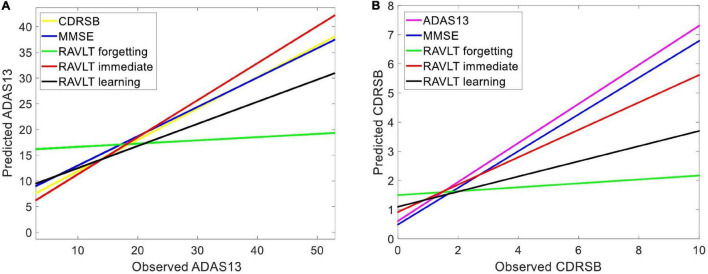
Predicting target neuropsychological scores based on other scores to find the association between neuropsychological tests. Various neuropsychological scores in three groups of subjects (i.e., HC, MCI-NC, and AD) were used as input features of an ANN to predict **(A)** ADAS13 and **(B)** CDRSB from normal aging to AD. Linear regressions for the predicted *vs*. observed values of ADAS13 and CDRSB are shown. *R*-squares of the linear regressions are listed in Table 2.

We used HC, MCI-NC, and AD datasets (by including all subjects with a stable state from normal aging to AD) to predict ADAS13 and CDRSB target scores based on other neuropsychological scores as input features of an ANN. Figure 4 shows linear regressions for the predicted *vs*. observed values of ADAS13 (left panel) or CDRSB (right panel) using MMSE, RAVLT Immediate, RAVLT Learning, and RAVLT Forgetting as an input feature of the ANN. In addition, we predicted the ADAS13 and CDRSB using one of these scores as input and another one as output of the ANN. It can be seen from Figure 4A and Table 2 that RAVLT Immediate provided the best prediction for the progression trend of ADAS13 (*R*-square = 0.80 and MSE = 33.10) from normal aging to AD that shows a high association between ADAS13 and RAVLT Immediate scores. The ADAS13 provided the best prediction (*R*-square = 0.80 and MSE = 1.15) in estimating the progression trend of CDRSB from normal aging to AD (see Figure 4B and Table 2). The CDRSB is commonly known as the severity indicator of AD and our results revealed an association between CDRSB and ADAS13. Notably, the RAVLT Forgetting had the worst performances in the prediction of ADAS13 and CDRSB (*R*-square = 0.25 and 0.13 and MSE = 97.65 and 3.02, respectively).

**TABLE 2:** *R*-square and mean square error (MSE) for modeling the progression trend of ADAS13 or CDRSB from normal aging to AD based on different inputs features (neuropsychological scores and single and multiple neuroimaging measures).

	Biomarker	ADAS13		CDRSB	
		*R*-square	MSE	*R*-square	MSE
Neuropsychological Tests	MMSE	0.77	38.12	0.78	1.25
	ADAS13	–	–	0.80	1.15
	CDRSB	0.82	37.23	–	–
	RAVLT learning	0.67	52.60	0.51	2.79
	RAVLT forgetting	0.25	97.65	0.13	3.02
	**RAVLT immediate**	**0.80**	**33.10**	**0.67**	**1.66**
sMRI [volumes of brain structures]	MIDTEMP	0.55	70.94	0.55	2.07
	Hippocampus	0.57	61.66	0.54	1.91
	**Entorhinal**	**0.67**	**58.27**	**0.65**	**1.87**
	Ventricles	0.27	81.32	0.28	2.81
	Fusiform	0.41	75.03	0.44	2.10
	ICV	0.12	113.71	0.15	3.44
PET	**FDG**	**0.66**	**48.33**	**0.70**	**1.64**
	AV45	0.39	75.00	0.41	2.40
sMRI + PET	**Entorhinal + FDG**	**0.74**	**37.61**	**0.80**	**1.35**
	Hippocampus + FDG	0.65	38.26	0.73	1.69
	Entorhinal + Hippocampus + FDG	0.69	40.95	0.79	1.55

*Note that the predictions with the best performance are shown in bold font.*

### Predicting Target Scores Based on Unimodal and Bimodal Neuroimaging Data

**FIGURE 5:**
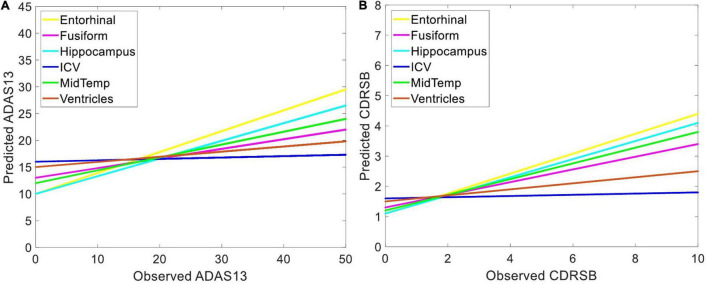
Predicting target neuropsychological scores based on structural biomarkers to find the best predictors of the scores. Volumes of different brain structures (extracted from sMRI) in three groups of subjects (i.e., HC, MCI-NC, and AD) were used as input features of an ANN to predict **(A)** ADAS13 and **(B)** CDRSB from normal aging to AD. Linear regressions for the predicted *vs*. observed values of ADAS13 and CDRSB are shown. *R*-squares of the linear regressions are listed in Table 2.

Independent ANN models were separately trained and tested using sMRI and/or PET input features to predict ADAS13 and CDRSB in order to find the best unimodal and bimodal neuroimaging features for this prediction. Prediction of the target scores based on six sMRI features (ICV and the volumes of five ROIs) are shown in Figure 5. As listed in Table 2 and shown in Figure 5, the volume of entorhinal cortex was the best predictor for ADAS13 (*R*-square = 0.67 and MSE = 58.27) and CDRSB (*R*-square = 0.67 and MSE = 1.87). The volume of the hippocampus was the second best predictor for ADAS13 (*R*-square = 0.57 and MSE = 61.66) and CDRSB (*R*-square = 0.54 and MSE = 1.91). These results are in line with the fact that the volumes of the hippocampus and entorhinal cortex are highly associated with memory function in the brain. As expected, the ICV was the worst predictor for both ADAS13 (*R*-square = 0.12 and RMSE = 113) and CDRSB (*R*-square = 0.15 and RMSE = 3.44).

**FIGURE 6:**
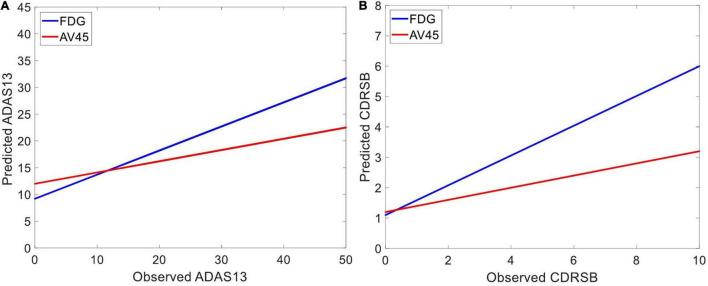
Predicting target neuropsychological scores based on PET biomarkers to the best predictors of the scores. The average FDG of the angular gyrus, temporal gyrus, and posterior cingulate and the average AV45 of frontal, anterior cingulate, precuneus, and parietal cortex in three groups of subjects (i.e., HC, MCI-NC, and AD) were used as input features of an ANN to predict **(A)** ADAS13 and **(B)** CDRSB from normal aging to AD. Linear regressions for the predicted *vs*. observed values of ADAS13 and CDRSB are shown. *R*-squares of the linear regressions are listed in Table 2.

**FIGURE 7:**
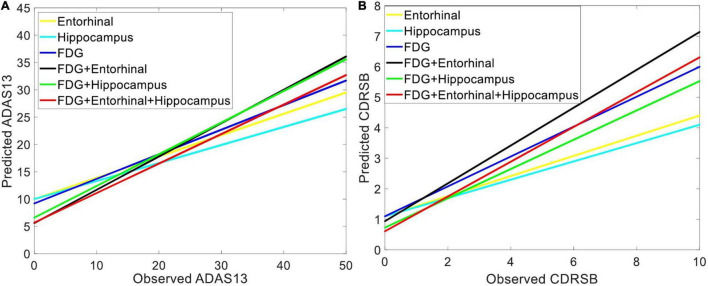
Comparing the unimodal and bi-modal predictors of the target neuropsychological scores. Combination of sMRI and PET features cortex in three groups of subjects (i.e., HC, MCI-NC, and AD) were used as input features of an ANN to predict **(A)** ADAS13 and **(B)** CDRSB from normal aging to AD. Linear regressions for the predicted *vs*. observed values of ADAS13 and CDRSB are shown. *R*-squares of the linear regressions are listed in Table 2.

Figure 6 shows the prediction results for ADAS13 and CDRSB based on PET biomarkers (FDG and AV45). Compared to AV45, the FDG had a superior association with both ADAS13 and CDRSB from normal aging to AD (*R*-square = 0.66 and 0.70 and MSE = 48.33 and 1.64 for ADAS13 and CDRSB, respectively). For prediction based on bimodal neuroimaging data, the following best biomarkers were selected based on the results of unimodal prediction: the volumes of the entorhinal cortex and hippocampus for sMRI and FDG for PET. Figure 7 shows the results of unimodal and bimodal prediction of ADAS13 and CDRSB. It can be seen from Figure 7 and Table 2 that integration of FDG and volume of entorhinal cortex had the best performance for both ADAS13 (*R*-square = 0.74 and MSE = 37.61) and CDRSB (*R*-square = 0.80 and MSE = 1.35). It is interesting to note that the combination of FDG and the volume of the entorhinal cortex was optimal for prediction of CDRSB and, especially, ADAS13. Adding the volume of the hippocampus to the two features did not improve performance of the prediction, likely due to the association between the volumes of the hippocampus and entorhinal cortex and a preference of the ANN model for having a smaller number of features to prevent overfitting.

### Predicting ADAS13 in Converting and Stable Stages of Alzheimer’s Disease

**FIGURE 8:**
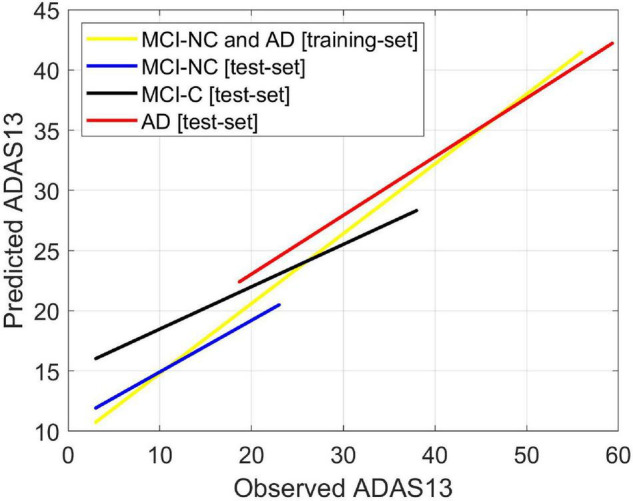
Predicting ADAS13 in converting and stable stages of AD by combination of the best bimodal biomarkers. The average FDG of the angular gyrus, temporal gyrus, and posterior cingulate and volume of the entorhinal cortex were used as input features of an ANN to predict ADAS13 and CDRSB in three stages from MCI to AD (i.e., MCI-NC, MCI-C, and AD). The ANN model was trained using the training-set of date in MCI-NC and AD groups. The trained model was then tested using the test-set data in the three groups (i.e., MCI-NC, MCI-C, and AD). Linear regressions for the predicted *vs*. observed value of ADAS13 are shown. The *R*-square of the linear regression model for the MCI-NC and AD [training-set] was 0.75. The *R*-squares of the linear regression models for the MCI-NC, MCI-C, and AD [test-set] were 0.43, 0.38, and 0.54, respectively.

We used FDG-PET and the volume of entorhinal cortex (as the best bimodal neuroimaging biomarkers) for predicting three stages from MCI to AD (i.e., MCI-NC, MCI-C, and AD). Our main goal was to investigate the ability of the bimodal neuroimaging biomarkers in modeling the target cognitive scores in the three stages. It is important to note that the baseline values of these neuroimaging biomarkers for MCI-NC and MCI-C groups were used in the prediction models while only patients in the latter group converted to AD in their follow-up visits. We trained ANN models to predict the two target scores in MCI-NC and AD groups using the FDG-PET and the volume of entorhinal cortex as input features. The MCI-NC and AD patients were chosen for training since they had a stable stage in their follow-up visits. Then the trained models were tested using the testing set of patients in MCI-NC, MCI-C, and AD groups. As shown in Figure 8, the predicted value of ADAS13 was different in the three groups and increasing trend for this value was observed from MCI-NC to MCI-C and from MCI-C to AD. We performed a one-way ANOVA to compare the predicted value of ADAS13 across three groups (MCI-NC, MCI-C, and AD). We performed the Shapiro–Wilk test for normality of the distributions of the predicted ADAS13 in the three groups and found that the distribution was significantly departed from normality (*P* < 0.007) in AD but was normal (*P* > 0.095) in MCI-NC and MCI-C. Therefore, we performed Kruskal-Wallis non-parametric ANOVA test and found a significant difference in predicted ADAS13 scores across the three groups (χ^2^ = 202.0, DF = 2, *P* < 0.0001). *Post hoc* comparisons using the Tukey HSD test indicated that the average rank of the predicted ADAS13 was significantly different in all combinations of two-group pairs (*P* < 0.0001). It is noteworthy that the predicted values of ADAS13 in the MCI-C group were significantly larger than that in the MCI-NC group, although the values of the input neuroimaging feature in baseline were used for both groups.

### Progression Trend of ADAS13 Based on Neuroimaging Biomarkers

**FIGURE 9:**
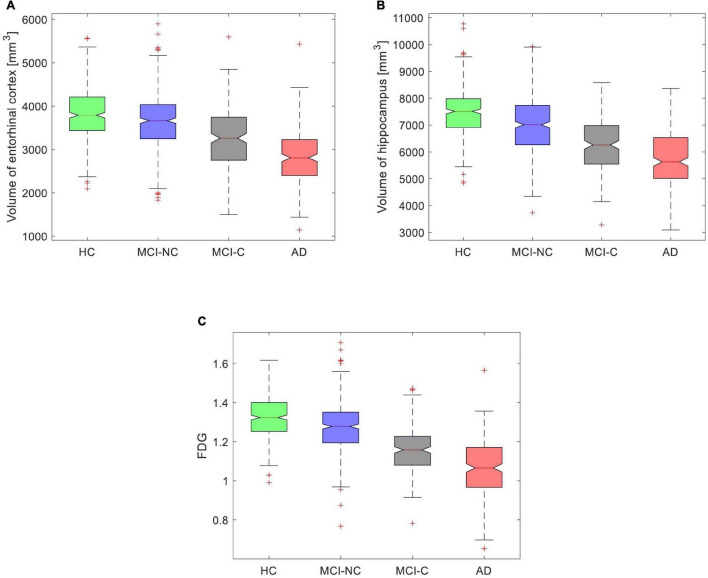
Box plot for values of **(A)** volume of the entorhinal cortex, **(B)** volume of the hippocampus, and **(C)** average FDG of the angular gyrus, temporal gyrus, and posterior cingulate in four groups (HC, MCI-NC, MCI-C, and AD).

The FDG-PET and volumes of the entorhinal cortex and hippocampus provided the best performance in predicting ADAS13 for PET and sMRI modalities. We used these features in all subjects (HC, MCI-NC, MCI-C, and AD) to model the progression trend of ADAS13 from normal aging to AD. As shown in Figure 9, there was a reducing trend in the values of the FDG-PET and volumes of the entorhinal cortex and hippocampus from normal aging to AD. We performed a one-way ANOVA to compare four groups (HC, MCI-NC, MCI-C, and AD) across the three neuroimaging features. Because of the non-Gaussian distributions of the FDG-PET and volume of hippocampus in AD based on the Shapiro–Wilk test (*W* = 0.99, *P* > 0.037), the Kruskal–Wallis non-parametric ANOVA test was used that revealed a significant effect for the volume of the entorhinal cortex (χ^2^ = 266.6, DF = 3, *P* < 0.0001), the volume of hippocampus (χ^2^ = 331.3, DF = 3, *P* < 0.0001), and the FDG-PET (χ^2^ = 428.7, DF = 3, *P* < 0.0001) across the four groups. *Post hoc* comparisons using the Tukey HSD test indicated that the average ranks of the volume of the entorhinal cortex, the volume of hippocampus, and the FDG-PET were significantly different in all combinations of two-group pairs (*P* < 0.0021).

**FIGURE 10:**
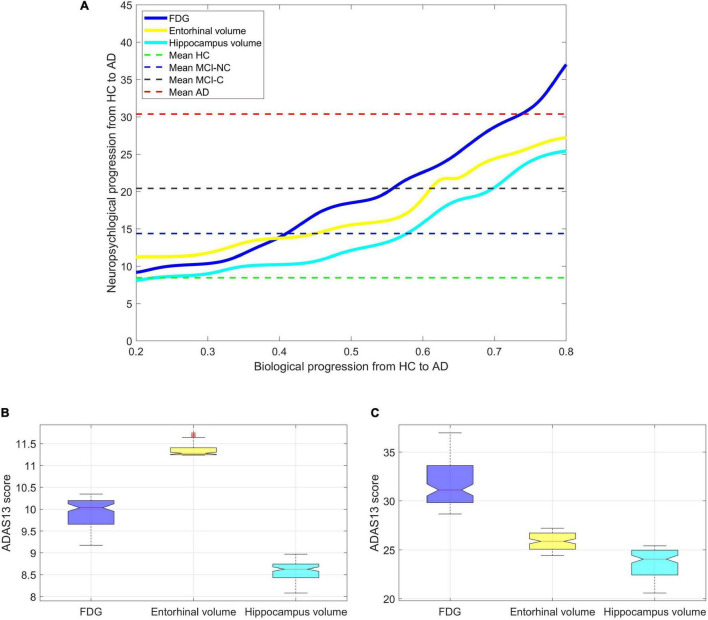
Finding the progression trend of ADAS13 score based on biological biomarkers from HC to AD. **(A)** The non-linear relationship between ADAS13 score and volumes of entorhinal cortex and hippocampus and the average FDG of the angular gyrus, temporal gyrus, and posterior cingulate. The values of the neuroimaging biomarkers (the *x*-axis in the figure) were normalized from 0 to 1 to represent the neurodegeneration trend from healthy aging to severe AD, respectively. **(B)** Values of the ADAS13 in mild neurodegeneration where the normalized values of the neuroimaging measures (i.e., FDG-PET and volumes of hippocampus and entorhinal cortex) were between 0.2 and 0.3. **(C)** Values of the ADAS13 in severe neurodegeneration where the normalized values of the neuroimaging measures were between 0.7 and 0.8.

Figure 10A shows a non-linear relationship between the values of the neuroimaging biomarkers and the predicted values of ADAS13 by the ANN in all subjects. The values of the neuroimaging biomarkers (the *x*-axis in Figure 10A) were normalized from 0 to 1 to represent the neurodegeneration trend from healthy aging to severe AD, respectively. Figures 10B,C show the distributions of the ADAS13 where the normalized values of the neuroimaging measures (i.e., FDG-PET and volumes of hippocampus and entorhinal cortex) were between 0.2 to 0.3 for mild neurodegeneration and between 0.7 and 0.8 for severe neurodegeneration, respectively. In mild neurodegeneration, the predicted values of ADAS13 by the non-linear model using FDG-PET were significantly smaller than that using the entorhinal cortex volume (*P* < 0.0001) based on the Wilcoxon rank-sum test. Conversely, in the severe neurodegeneration, the predicted values of ADAS13 by the non-linear model using FDG were significantly larger than that using the entorhinal cortex volume (*P* < 0.0001). Nevertheless, the predicted values of ADAS13 by the non-linear model using the hippocampus volume were significantly smaller than that using FDG and the entorhinal cortex in both mild and severe neurodegenerations. It is noteworthy that we performed the non-parametric Wilcoxon rank-sum test instead of a *t*-test here since the distributions of the three neuroimaging measures were significantly departed from normality (*P* < 0.0035) based on the Shapiro–Wilk test.

Our results indicate that the volume of the entorhinal cortex may be a better biomarker for identification of the early stage of AD compared to the FDG-PET. However, in the severe stage of neurodegeneration, the predicted value of ADAS13 based on the FDG-PET is larger than that based on the volume of the entorhinal cortex, which indicates that the former neuroimaging may be a better biomarker in the identification of late stage AD.

## Discussion

We utilized the ANN to investigate the association between the sMRI and PET biomarkers with two target neuropsychological scores (i.e., ADAS13 and CDRSB) from normal aging to AD. Using data in 951 subjects (341 HC, 393 MCI-NC, and 217 AD), our results revealed that the RAVLT Immediate, among five different neuropsychological tests (e.g., MMSE), provided the best prediction for the progression trend of ADAS13. Compared to other neuropsychological tests, however, the ADAS13 was the best predictor for the CDRSB score. Two remarks can be inferred from Figure 4 by exploring the association between ADAS13/CDRSB and other neuropsychological scores. First, the RAVLT Immediate was the best predictor for ADAS13 and the ADAS13 was the best predictor for the CDRSB. In addition, we observed a strong association between ADAS13, CDRSB, RAVLT Immediate, and MMSE. Second, the RAVLT Forgetting score is not correlated with ADAS13 or CDRSB scores, and, thus, the former score may provide independent information about the cognitive declines in AD compared to the latter scores. These observations indicate that the RAVLT tests should be integrated with other tests to assess different domains of cognitive declines in AD (Tosi et al., 2020). Previous studies reported that the RAVLT test can assist in identifying patients with subjective memory complaints who progress to AD, and this test can be used to differentiate MCI from normal aging (Estévez-González et al., 2003).

We found an association between neuroimaging (sMRI and PET) biomarkers and the target cognitive scores (i.e., ADAS13 and CDRSB) across a wide range of cognitive declines from normal aging to AD (Table 2). Uni-modal prediction of the target scores based on six sMRI features provided a competitive performance, but the entorhinal volume (followed by the hippocampus volume) outperformed other features (Figure 5). For PET features, results in Figure 6 show that the FDG outperformed AV45 for association with ADAS13 and CDRSB in three groups of subjects (i.e., HC, MCI, and AD). Our results for association of cognitive declines in AD (measured by the ADAS13 and CDRSB scores) with the volumes of the entorhinal cortex and hippocampus (extracted from sMRI) and the average FDG of the angular gyrus, temporal gyrus, and posterior cingulate are in line with previous studies (Wang et al., 2018; Prosser et al., 2020). The hippocampus is the core of the neural memory system and the entorhinal cortex is the hub for the widespread network for memory, navigation, and perception of time (O’Keefe, 1976; Eichenbaum et al., 2007). Atrophy of the hippocampus and entorhinal cortex have been highly associated with AD progression (Jessen et al., 2006; Devanand et al., 2007, 2012; Stoub et al., 2010). FDG-PET is also a well-known technique to identify the brain glucose metabolism which is mainly determined by synaptic activity in the brain. Previous studies reported capability of the FDG-PET in identification of neurocognitive declines in AD (Liu et al., 2018; Ding et al., 2019). It has been reported that AD patients have significantly reduced glucose metabolism in the angular gyrus, temporal gyrus, and posterior cingulate (Hunt et al., 2007). These areas are involved in memory deficit in the early stages of AD. A severe hypometabolic pattern has been associated with awareness of memory deficit (Nobili et al., 2010). It is well known that the posterior cingulate cortex and entorhinal cortex are involved in memory retrieval and self-referential processes (Izquierdo et al., 1997; Maddock et al., 2001), and a strong relationship between atrophy in these cortices with cognitive declines in AD has been reported (Mosconi et al., 2004; Hirao et al., 2006).

By comparing performances of the unimodal neuroimaging features for prediction of the target scores (i.e., ADAS13 and CDRSB), we found that the FDG-PET outperformed the six sMRI features, including the volumes of the entorhinal cortex and hippocampus. Furthermore, the bimodal prediction results (Table 2 and Figure 6) show that integrating two modalities (i.e., sMRI and PET) outperformed the unimodal approach. A previous study compared performance of FDG-PET and voxel-based morphometry (VBM) on MRI for classification of mild AD in unimodal and bimodal approaches (Kawachi et al., 2006). Results of this study revealed that the combination of two modalities provided a higher diagnostic accuracy (94%) compared to the unimodal approach based on FDG-PET (89%) or VBM-MRI (83%). Results of this study are in line with our findings showing superior performance of the bimodal approach compared to a unimodal approach. However, it is noteworthy that no study, to our knowledge, has yet investigated integrating PET and sMRI for predicting the ADAS13 and CDRSB across a wide range of cognitive decline from normal aging to severe AD. Previous studies investigated the relationship between the neuropsychological assessments and neuroimaging biomarkers (Godbolt et al., 2005; Musicco et al., 2009; Ito et al., 2011), and most of them utilized a single modality (typically sMRI) approach for this investigation (Frisoni et al., 2002, 2010; Apostolova et al., 2006). The structural-based biomarkers, such as gray matter volume and cortical thickness, have been utilized to find the association between neuropsychological scores and brain atrophy in AD (Frisoni et al., 2002; Zhou et al., 2013).

Our results in Figure 8 confirmed that integration of the entorhinal volume with the average FDG of the angular gyrus, temporal gyrus, and posterior cingulate was capable of predicting ADAS13 in the MCI-C, MCI-NC, and AD groups. Interestingly, the predicted trends of the ADAS13 score in the three groups show that the MCI-C group is an intermediate stage between MCI-NC and AD groups (Borroni et al., 2006). Another observation in Figure 8 for MCI-C subjects is that the ANN algorithm predicted a larger value for the ADAS13 score than its real value. This observation indicates that the neuroimaging biomarkers may be more sensitive in identification of the early stage of AD as the predicted value of ADAS13 by these biomarkers is larger than its real value in MCI-C patients. It has been reported that there is a long asymptomatic period (up to two decades) between the onset of brain changes and reaching an endpoint with the earliest development of clinical symptoms of AD (Mesulam, 1999; Perry and Hodges, 1999). In fact, many patients reach their end of life without developing fully characterized AD (Sperling et al., 2011). Therefore, current diagnostic criteria for AD depends significantly on the imaging biomarkers of the AD pathologies (DeKosky and Marek, 2003; Johnson et al., 2012; Bonifacio and Zamboni, 2016; Jagust, 2018) as the biological processes underlying AD may occur while the patient’s cognitive scores are still in MCI stage.

We investigated a relationship between the brain structural and functional neurodegeneration and the ADAS13 score from normal aging to severe AD (Figure 10). The curves related to the atrophy of the hippocampus and entorhinal cortex showed a plateau at the severe stage of AD (i.e., x > 0.75 in Figure 10) while the FDG-PET curve did not show this plateau. This observation indicates that the volumes of the entorhinal cortex and hippocampus have less sensitivity than the FDG-PET to model progression of AD in the severe stage of this disease. By comparing the structural atrophy in healthy aging subjects and patients with a very MCI, we found that the average volumes of hippocampus and entorhinal cortex across MCI-NC patients were 6.1 and 3.7%, respectively, smaller than that across HC subjects (Figure 9). These results are in agreement with previous studies that age-related atrophy in the medial temporal lobes occurs with larger hippocampal decline than the entorhinal cortex (Raz et al., 2004; Rodrigue and Raz, 2004). We also found that the average ADAS13 in MCI-NC patients corresponded with a larger atrophy in the hippocampus compared to the entorhinal cortex (57.32% *vs*. 46.73%, Figure 10). Considering that the ADAS score measures the severity of cognitive and non-cognitive dysfunction from mild to severe AD (Rosen et al., 1984), our results support a more important role of the hippocampus in this dysfunction compared to that of the entorhinal cortex. Specific to memory decline, Jessen et al. (2006) reported that the significantly affected entorhinal cortex causes slight memory dysfunction at the earliest clinically detectable stage of AD when patients experience worsening memory and the hippocampus is significantly affected with disease progression. Our results revealed that the volumes of hippocampus and entorhinal cortex reduced approximately 25% from a mild to severe stages of AD by comparing the average volumes of these structures in MCI-C and AD patients (Figure 9). Referring to the large atrophy of these structures in the mild to severe stages of AD, these two biomarkers showed a plateau for modeling ADAS13 in these stages in Figure 10. Based on the estimated ADAS13 curves corresponding to the volumes of hippocampus and entorhinal cortex, it can be inferred that these structural biomarkers had a good sensitivity in identification of the early stage of AD and they may not be sensitive for identification of the severe stage of AD. This observation is consistent with a general understanding of AD progression and the different stages of progression of dementia (Kung et al., 2021).

Our results revealed an association between FDG-PET and ADAS13/CDRSB. This observation shows that the glucose metabolism can measure alteration of cognition and functional ability in patients with MCI and AD. We also found that FDG-PET can track the AD progression and has a potential to be used as a clinically helpful measure of cognitive decline, particularly in MCI and AD patients. Previous studies reported that a lower FDG-PET at baseline has an association with greater longitudinal cognitive decline in AD (Alexander et al., 2002; Ottoy et al., 2019) and FDG-PET at a pre-dementia stage (MCI) has higher sensitivity to subsequent decline than neuropsychological tests (Chételat et al., 2005).

In this study, we used ANNs for quantitative analysis of the AD progression. This analysis can be used as a guide to help in the evaluation of different AD study designs, as well as to understand the complex relationship between various factors such as neuropsychological scores and imaging data. Establishing the relationships between neuropsychological scores and imaging data can help identify potential surrogates of clinical outcome and may guide to design future clinical trials.

## Data Availability Statement

Publicly available datasets were analyzed in this study. This data can be found here: https://tadpole.grand-challenge.org.

## Ethics Statement

Ethical review and approval was not required for the study on human participants in accordance with the local legislation and institutional requirements. The patients/participants provided their written informed consent to participate in this study.

## Author Contributions

SH: preparing required data, data analysis, statistical analysis, and drafting and revision of the manuscript. AB-F: study design and conceptualization, data analysis and interpretation of the results, and drafting and revision of the manuscript. All authors contributed to the article and approved the submitted version.

## Conflict of Interest

The authors declare that the research was conducted in the absence of any commercial or financial relationships that could be construed as a potential conflict of interest.

## Publisher’s Note

All claims expressed in this article are solely those of the authors and do not necessarily represent those of their affiliated organizations, or those of the publisher, the editors and the reviewers. Any product that may be evaluated in this article, or claim that may be made by its manufacturer, is not guaranteed or endorsed by the publisher.

## Footnotes

1
https://tadpole.grand-challenge.org

2
adni.loni.usc.edu

3 Data used in the preparation of this article were obtained from the Alzheimer’s Disease Neuroimaging Initiative (ADNI) database (adni.loni.usc.edu). The ADNI was launched in 2003 as a public-private partnership, led by Principal Investigator Michael W. Weiner, MD. The primary goal of ADNI has been to test whether serial magnetic resonance imaging (MRI), positron emission tomography (PET), other biological markers, and clinical and neuropsychological assessment can be combined to measure the progression of mild cognitive impairment (MCI) and early Alzheimer’s disease (AD). For up-to-date information, see www.adni-info.org

4
http://adni.loni.usc.edu/methods/mri-analysis/mri-pre-processing

5
http://adni.loni.usc.edu/methods/pet-analysis/pre-processing
